# Comparative Assessment of the Sensitivity of Ten Commercial Rapid Diagnostic Test Kits for the Detection of *Plasmodium*

**DOI:** 10.3390/diagnostics12092240

**Published:** 2022-09-16

**Authors:** Mathieu Gendrot, Marylin Madamet, Isabelle Fonta, Nicolas Benoit, Rémy Amalvict, Joel Mosnier, French National Reference Centre for Imported Malaria Study Group, Bruno Pradines

**Affiliations:** 1 Unité Parasitologie et Entomologie, Département de Microbiologie et Maladies Infectieuses, Institut de Recherche Biomédicale des Armées, 13005 Marseille, France; 2 Aix Marseille University, IRD, AP-HMM, SSA, VITROME, 13005 Marseille, France; 3 IHU Méditerranée Infection, 13005 Marseille, France; 4 Centre National de Référence du Paludisme, 13005 Marseille, France

**Keywords:** malaria, *Plasmodium falciparum*, diagnosis, rapid diagnostic test, RDT, LDH, HRP2

## Abstract

Malaria is one of the most common tropical diseases encountered by members of the French military who are deployed in operations under constrained conditions in malaria-endemic areas. Blood smear microscopy—the gold standard for malaria diagnosis—is often not available in such settings, where the detection of malaria relies on rapid diagnostic tests (RDTs). Ten RDTs (from Biosynex, Carestart, Humasis, SD Bioline, and CTK Biotech), based on the detection of the *Plasmodium falciparum* histidine-rich protein 2 (HRP2) or lactate dehydrogenase (pLDH, PfLDH, or PvLDH), were assessed against 159 samples collected from imported malaria cases, including 79 *P. falciparum*, 37 *P. vivax*, 22 *P. ovale*, and 21 *P. malariae* parasites. Samples had been previously characterised using microscopy and real-time PCR. The overall sensitivities for the *Plasmodium* test ranged from 69.8% (111/159) to 95% (151/159). There was no significant difference for the specific detection of *P. falciparum* (96.2% to 98.7%, *p* = 0.845). No significant difference was found between sensitivities to *P. vivax* by pan LDH or pvLDH (81.1% (30/37) to 94.6% (35/37) (*p* = 0.845)). Some of the RDTs missed most of *P. ovale* and *P. malariae*, with sensitivities for all RDTs ranging respectively from 4.5% (1/22) to 81.8% (18/22) and 14.3% (3/21) to 95.2% (20/21). Carestart Malaria Pf/Pan (pLDH) Ag G0121, a pLDH-based RDT (PfLDH and pLDH), showed the highest sensitivities to *P. falciparum* (98.7%, 78/79), *P. vivax* (94.6%, 35/37), *P. ovale* (81.8%, 18/22), and *P. malariae* (95.2%, 20/21) and meets the requirements for military deployments in malaria-endemic areas.

## 1. Introduction

Malaria remains a major problem for human health and in particular for the French military, which encountered more than 6000 cases between 2000 and 2015 [1,2]. Sixty-two percent of these cases occurred in endemic areas. Soldiers can be deployed far from health facilities or medical personnel and, therefore, it is imperative to ensure the most effective diagnostic tools adapted to their restricted conditions are available.

Members of the French military are deployed in parts of Africa where *P. falciparum* is predominant, but also in areas of the continent where the transmission of *P. ovale* is reported, such as Côte d’Ivoire, Gabon, and Senegal [3,4,5]. Moreover, many military operations are performed every year in French Guiana, where *P. vivax* is predominant [6]. Between 2000 and 2015, 6468 cases of malaria were reported in the French Armed Forces, with 60.7% of *P. falciparum*, 29.0% of *P. vivax*, 7.2% of *P. ovale*, and 1.8% of *P. malariae* [1]. Consequently, it is important to identify RDTs which are effective at detecting all species.

The rapid diagnosis of malaria is essential due to the potential fast progression from uncomplicated malaria to severe malaria and death, especially in non-immune patients without appropriate treatment (such as the use of ACTs) [7,8]. In mainland France, legally the diagnosis has to be performed within two hours after reception of the sample [9]. Microscopic diagnosis, based on the direct observation of the asexual stages of the parasite on a thin or thick blood smear, is the standard method. However, these microscopic techniques require specific equipment and trained personnel and are time consuming. Furthermore, the main limitation of the diagnosis is that these microscopic methods are almost never available in military deployment settings and locations with limited resources.

Malaria rapid diagnostic tests (RDTs), based on immunochromatographic techniques, are complementary techniques used in laboratory settings in mainland France. RDTs are fast (results within 15–30 min of migration), and relatively easy to use and interpret [10]. However, the results need to be interpreted by trained personnel. RDTs are adapted for use in field conditions. However, their accuracy varies depending on the brand used [10]. Currently, more than 200 different malaria RDTs are commercially available. Most of them are designed to identify *Plasmodium falciparum*-specific proteins and target either the *P. falciparum* histidine-rich protein 2 (HRP2) or the *P. falciparum* lactate dehydrogenase (PfLDH). RDTs can detect other pan-malaria (genus) antigens, such as pLDH or pALD (aldolase). Some kits can detect the specific protein *P. vivax* lactate dehydrogenase (PvLDH). The users of the RDT and their institution must select the most efficient kit according to their needs (sensitivity, specificity, stability, storage conditions). Moreover, the availability of a complete kit is essential. In each bag, there is an RDT, a solvent vial, an alcohol pad, and a needle. This helps to avoid the loss of the solvent vial or other essential elements, which are often sold in a single container for a whole box of 20–25 RDTs.

The aim of our study was to compare the sensitivity of different RDT kits referenced in the World Health Organization (WHO) RDT testing report [11] and marketed in France, in order to select the best kit for the detection of *plasmodium* in an endemic area within a constrained environment. The thin blood smear, the gold standard method, and real time PCR—the most sensitive and specific method—were used in parallel to check the validity of the results rendered by the RDTs. We hope this study will change the policy on the use of RDTs in the French military, in favour of a more sensitive ‘lab-in-a- kit’RDT package.

## 2. Materials and Methods

### 2.1. Sample Collection

The isolates were collected from patients hospitalised in several civilian and military hospitals belonging to the French National Reference Centre for Imported Malaria network (Aix-en-Provence, Bordeaux, Lyon, Marseille, Montpellier, Nice, Toulon, Toulouse and Valence) between September 2018 and February 2019. The patients presented with imported malaria from a malaria-endemic country and their samples were sent to the French National Reference Centre for Malaria (CNR) (Institut de RechercheBiomédical des Armées, IHU Méditerranée Infection, Marseille). Whole blood was received in EDTA Vacutainer tubes after being transported from hospitals in the CNR. The samples were stored and transported at 4 °C from the moment of sampling until we receive the samples. A total of 159 samples were evaluated including 79 *P. falciparum* (parasitaemia ranging from 0.001% to 21%), 37 *P. vivax* (parasitaemia ranging from 0.001% to 0.6%), 22 *P. ovale* (parasitaemia ranging from 0.001% to 0.6%), and 21 *P. malariae* (parasitaemia ranging from 0.001% to 0.4%).

### 2.2. Malaria Diagnostic Techniques

Parasite species and parasitaemia assessments were carried out by certified operators on thin blood smears that were stained with eosin and methylene blue using an RAL^®^ kit(Réactifs RAL, Paris, France). Parasitaemia was computed on a minimum of 10 microscopic fields.

DNA extraction for each sample was performed using the QIAamp^®^ DNA Mini kit according to the manufacturer’s recommendations (Qiagen, Hilden, Germany).

Real time PCR was performed using the LightCycler 2.0 (Roche Group, Switzerland), as previously described [12]. Briefly, the aquaglyceroporine of *P. falciparum* (NCBI:AJ413249), enoyl-acyl carrier protein reductase of *P. vivax*(NCBI:AY423071)*,* circumsporozoite (NCBI:S69014), ookinete surface protein 25 (NCBI:AB074976) of *P. ovale* were amplified with Roche LightCyclerTaqMan master (Roche Diagnostics, Meylan, France).

RDTs were selected according to the last WHO report on the performance of malaria RDTs on *P. falciparum* but also *P. vivax* parasites [9]. Members of the French military are exposed to *P. vivax* in French Guiana where *P. vivax* is predominant. The first nine RDT kits approved and marketed in France were selected from the list of WHO validated tests. Among these nine RDTs, three were selected for their specificity to detect *P. vivax* parasites. The main criteria for selection were market availability and the ‘lab-in-a-kit’format. The RDT kit most commonly used by the French army health service at the time of the study (SD Malaria Ag Pf/Pan (05FK63), SD BiolineAlere Abbott) was added to the nine RDTs. The 10RDTs are presented in Table 1. RDTs were used as recommended by each manufacturer.

### 2.3. Statistics

The statistics were collated using an Excel spreadsheet and Charles Zaiontz’s Real Statistics add-on (https://www.real-statistics.com/, accessed on 14 September 2022).

## 3. Results

All 159 samples were identified by thin blood smears and confirmed by real time PCR with a 100% match. Table 2 shows the sensitivities for each RDT.

The sensitivities for all species of *Plasmodium* ranged from 69.8% to 95%. The difference between the 10 RDTs was significant (*p* < 0.001, Pearson’s chi-squared test). The RDTs with the highest sensitivity to *Plasmodium* spp. (Carestart G0121) detected significantly more parasites than RDTs with a sensitivity below 86.8% (*p* = 0.018, Fisher exact test).

No significant difference was found between sensitivities to *P. vivax* by pan LDH or pv-specific LDH, which ranged from 81.1% to 94.6% (*p* = 0.845, Pearson’s chi-squared test).

Some of the RDTs (SD Bioline 05FK63, AMAL-7025, Onesite R0113C, Palutop 4+ Optima) missed most of the *P. ovale* parasites, with sensitivities for all RDTs ranging from 4.5% to 81.8% (*p* < 0.001, Pearson’s chi-squared test).

The sensitivities for detection of *P. malariae* ranged from 14.3% to 95.2% (*p* < 0.001, Pearson’s chi-squared test). The RDTs which missed the *P. malariae* parasites were the same, with low sensitivities to *P. ovale*.

There was no significant difference for the specific detection of *P. falciparum* (96.2% to 98.7%, *p* = 0.845, Pearson’s chi-squared test). There was no cross detection between parasite species and the 10 different RDTs. However, on Palutop 4+, there were 15 out of 79 *P. falciparum* isolates that were positive on the “*P. vivax* LDH” band of the RDT (parasitaemia ranging from 0.25% to 12.7%). This has already been observed in the past, when Palutop 4+ was used in French Guyana, when sometimes when there is high parasitaemia or high levels of circulating HRP2, the *P. falciparum* HRP2 band is saturated and the *P. vivax* LDH turns positive. The two other RDTs with a *P. vivax* LDH band showed no cross reaction (Carestart and Humasis).

## 4. Discussion

It is vital for military personnel who are deployed with limited resources often without health personnel or reliable diagnostic means to be able to quickly detect the presence of malaria parasites. When there is no health facility nearby, ACT treatment must be administered directly in the field. In these conditions, clinical symptoms combined with the use of an RDT are the only clues the operator will have to diagnosis malaria. More than 200 malaria RDTs are currently commercially available worldwide and over 80 kits fulfil the criteria required by the WHO (performance, stability, ease of use) [13]. Several RDTs are available in France, and it is the responsibility of the health service of the French Army to select the best RDT kit according to their needs.

The 10RDTs were efficient in the detection of *P. falciparum* parasites with sensitivities ranging from 96.2% to 98.7%, while no differences were found in terms of specificity. Carestart Malaria Pf/Pan (pLDH) Ag (G0121) was the only one of the 10 RDTs which relied on specific pfLDH for the detection of *P. falciparum*. The nine other RDTs relied on HRP2 detection. The majority of the RDTs are designed to identify HRP2. Only 11/89 tests, based on the detection of pfLDH or both pfLDH-HRP2, met the WHO requirements for RDT procurement [14]. Previous pLDH-based RDTs—more particularly, Optimal-IT—seemed to be less sensitive than HRP2-based RDTs [15,16,17]. The more recent pLDH-based RDTs showed a higher sensitivity to *P. falciparum* [18,19] than previously, therefore they could be used for normal RDT usage. The present study did not reveal detection differences between target molecules of HRP2 and LDH in the 10RDTs. In the context of *P. falciparum* parasites lacking the *pfhrp2* gene [20], Carestart Malaria Pf/Pan (pLDH) Ag G0121 would address this issue. The high prevalence of *pfhrp2*-deleted mutants associated with false-negative results with HRP2-based RDTs have been reported in various countries (Peru, Eritrea, Ghana, Rwanda) [20,21,22,23]. Moreover, the proportion of false-positives in pfLDH-based RDTs after antimalarial treatment was very low in comparison with HRP2-based RDTs, due to the persistence of HRP2 antigens for more than a month [24]. The detection of PfLDH antigens can be used for monitoring antimalarial drug efficacy. This helps to select the appropriate RDT in areas harbouring deleted HRP2 parasites.

Five RDTs (SD Bioline Ag Pf/Pan 05FK63, Malaria Pf/Pan Antigen, Test AMAL-7025, Onesite Malaria Pf/Pan Ag R0113C, Palutop 4+ Optima and Carestart Malaria Pf/VOM combo G0171) showed global sensitivity to *Plasmodium spp* which was under 90% (*p* < 0.018 compared to Carestart Malaria Pf/Pan (pLDH) Ag G0121). These RDTs missed many *P. ovale* (4.5% to 59.1%) and *P. malariae* (14.3% to 71.4%). Some RDTs, such as Palutop 4+ Optima and SD Bioline Ag Pf/Pan 05FK63, were already known for their poor efficiency at detecting *P. ovale* and *P. malariae* [25,26]. The five Carestart RDTs were significantly more efficient at detecting *P. ovale* (59.1% to 81.8%) and *P. malariae* (71.4% to 95.2%) than the other RDTs (4.5% to 9.1%, *p*< 0.001, Fisher exact test and 14.3% to 33.3%, *p*< 0.029, respectively). Carestart Malaria Pf/Pan (pLDH) Ag G0121 was the most efficient at detecting *P. ovale* (81.8%) and *P. malariae* (95.2%).

There was no significant difference in the detection of *P. vivax* between the 10RDTs (81.1% to 94.6%, *p* = 0.845, Pearson’s chi-squared test). Three RDTs, Malaria Pf/Pv Antigen Test AMFV-7025, Carestart Malaria Pf/Pv (HRP2/pLDH) Ag combo G0161 and Palutop 4+ Optima, specifically identified *P. vivax* (one specific band on RDTs) due to the detection of PvLDH (sensitivities from 83.5% to 91.9%) but were not more efficient at detecting *P. vivax* parasites than pLDH-based RDTs. The only advantage of these three RDTs would be that they could be used to detect *P. falciparum-vivax* mixed infections in areas where *P. vivax* was predominant and where there was a very low transmission of *P. ovale* and *P. malariae* parasites, such as in French Guiana and the Republic of Djibouti [6,27,28]. In the absence of the specific detection of *P. vivax*, only *P. falciparum* will be identified (one band-HRP2 or -PfLDH and one band-pLDH). If the RDT misses the *P. vivax* parasite, the patient will still be treated for uncomplicated *falciparum* malaria. This treatment includes artenimol-piperaquine or artemether-lumefantrine, which are the first-line curative treatments which are recommended in France for uncomplicated imported *P. falciparum* and non-*falciparum* malaria [29]. The same recommendations have been adopted by the French armed forces in their military operations and deployments to malaria-endemic areas. The specific radical cure for *P. vivax* using primaquine is prescribed during a resurgence in the absence of G6PD deficiency, when only the *P. vivax* parasites are detected.

The main limitation of this study was the low number of samples, although 80 non-falciparum samples were tested. There was no issue with sample preservation due to the short timescale within which the samples were sent to the CNR and the fact that the transport was maintained at 4 °C, as controlled by temperature tracker in each package.

## 5. Conclusions

This study showed that some RDTs missed the detection of *P. ovale* and *P. malariae* and would therefore not be appropriate for either malaria diagnosis in the French armed forces or for cases of imported malaria. Carestart Malaria Pf/Pan (pLDH) Ag G0121, a non HRP2-based RDT, showed the highest sensitivities to *P. falciparum* (98.7%), *P. vivax* (94.6%), *P. ovale* (81.8%), and *P. malariae* (95.2%). This RDT meets the requirements of the French armed forces with regards to their military deployments in malaria-endemic areas: the time-to-results is rapid, it does not require extensive training or specific equipment, it is efficient for all *Plasmodium* parasites, and it can be used worldwide, even in a context of *pfhrp2* deletions. This RDT also meets all the criteria for the diagnosis of imported malaria in non-malaria-endemic countries. As some of those RDTs meet the requirements of the French military forces in their deployments, they also could be useful for other applications linked with malaria diagnosis, such as in low-income countries where RDTs are used as a major diagnostic tool.

## Figures and Tables

**Table 1:** Characteristics of the 10 malaria rapid diagnostic tests (RDT).

Identification	Company	Antigens (Specificity)	Batch Number (Lapsing)
SD Malaria Ag Pf/Pan (05FK63)	SD BiolineAlere Abbott	HRP2 (Pf) pLDH (Pf + Pv + Po + Pm)	05EDD002A (10 January 2020)
Malaria Pf/PAN Antigen Test (AMAL-7025)	Humasis, Launch Diagnostics	HRP2 (Pf) pLDH (Pf + Pv + Po + Pm)	MAL7040 (11 December 2019)
Malaria Pf/Pv Antigen Test (AMFV-7025)	Humasis, Launch Diagnostics	HRP2 (Pf) LDH (Pv)	MFV8003 (15 May 2020)
OnesiteMalaria Pf/Pan Ag Rapid Test (R0113C)	CTK Biotech, Eurobio	HRP2 (Pf) pLDH (Pf + Pv + Po + Pm)	F1114N3I03 (14 November 2019)
Palutop4+ Optima	Biosynex	HRP2 (Pf) LDH (Pv) pLDH (Pf + Pv + Po + Pm)	[91226] (December 2019)
CareStart Malaria pLDH Pf/Pan (Med G0121)	Medequip	pLDH (Pf) pLDH (Pf + Pv + Po + Pm)	ML 18H61 (July 2020)
CareStart Malaria HRP2/pLDH Pf/Pan Combo (Med G0131)	Medequip	HRP2 (Pf) pLDH (Pf + Pv + Po + Pm)	MF 18F63 (November 2020)
CareStart Malaria HRP2/pLDH Pf/VOM Combo (Med G0171)	Medequip	HRP2 (Pf) LDH (Pv + Po + Pm)	MW 18G61 (June 2020)
CareStart Malaria Screen (Med G0231)	Medequip	HRP2/LDH (Pf) pLDH (Pf + Pv + Po + Pm)	MA 18G61 (June 2020)
CareStart Malaria HRP2/pLDH Pf/Pv Combo (Med G0161)	Medequip	HRP2 (Pf) LDH (Pv)	MV 18G62 (June 2020)

HRP2: Histidine Rich Protein 2, (p)LDH: (pan) Lactate dehydrogenase, Pf: *P. falciparum*; Pv: *P. vivax*; Po: *P. ovale*; Pm: *P. malariae.*

**Table 2:** Sensitivity of 10 rapid diagnostic test (RDT) kits against 159 samples including 79 *P. falciparum* (Pf), 37 *P. vivax* (Pv), 22 *P. ovale* (Po), and 21 *P. malariae* (Pm).

RDT Brand	Antigen Detection	Sensitivity *P. falciparum*	Sensitivity *P. vivax*	Sensitivity *P. ovale*	Sensitivity *P. malariae*	Sensitivity to All *Plasmodium* Species
SD Malaria Ag Pf/Pan (25FK63)	HRP2 (Pf)	96.2% (76/79)				69.8% (111/159)
	pLDH (Pf + Pv + Po + Pm)	64.6% (51/79)	81.1% (30/37)	4.5% (1/22)	19.0% (4/21)	
Malaria Pf/Pan Antigen Test (AMAL-7025)	HRP2 (Pf)	96.2% (76/79)				69.8% (111/159)
	pLDH (Pf + Pv + Po + Pm)	77.2% (61/79)	83.8% (31/37)	4.5% (1/22)	14.3% (3/21)	
Onesite Malaria Pf/Pan Ag Rapid Test (R0113C)	HRP2 (Pf)	97.5% (77/79)				71.1% (113/159)
	pLDH (Pf + Pv + Po + Pm)	72.2% (57/79)	83.8% (31/37)	9.1% (2/22)	14.3% (3/21)	
Carestart Malaria Screen (Med G0231)	HRP2/LDH (Pf)	98.7% (78/79)				91.8% (146/159)
	pLDH (Pf + Pv + Po + Pm)	88.6% (70/79)	86.5% (32/37)	77.3% (17/22)	90.5% (19/21)	
Carestart Malaria Pf/Pan (pLDH) Ag (Med G0121)	LDH (Pf)	98.7% (78/79)				95.0% (151/159)
	pLDH (Pf + Pv + Po + Pm)	89.9% (71/79)	94.6% (35/37)	81.8% (18/22)	95.2% (20/21)	
Carestart Malaria Pf/Pan (HRP2 /pLDH) Ag Combo (Med G0131)	HRP2 (Pf)	98.7% (78/79)				92.5% (147/159)
	pLDH (Pf + Pv + Po + Pm)	84.8% (67/79)	89.2% (33/37)	77.3% (17/22)	90.5% (19/21)	
Carestart Malaria Pf/VOM (HRP2 /pLDH) Combo (Med G0171)	HRP2 (Pf)	98.7% (78/79)				86.8% (138/159)
	LDH (Pv + Po + Pm)		86.5% (32/37)	59.1% (13/22)	71.4% (15/21)	
Carestart Malaria Pf/Pv (HRP2 /pLDH) Ag Combo (Med G0161)	HRP2 (Pf)	98.7% (78/79)				
	LDH (Pv)		86.5% (32/37)			
Malaria Pf/Pv Antigen Test (AMFV-7025)	HRP2 (Pf)	96.2% (76/79)				
	LDH (Pv)		83.8% (31/37)			
Palutop 4+ Optima	HRP2 (Pf)	98.7% (78/79)				75.5% (120/159)
	LDH (Pv)		91.9% (34/37)			
	pLDH (Pf + Pv + Po + Pm)	84.8% (67/79)	89.2% (33/37)	4.5% (1/22)	33.3% (7/21)	

## Data Availability

The datasets analysed in this study are available from the corresponding author on reasonable request.

## Abbreviations

Aldolase: ALD; Deoxyribonucleic acid: DNA; Histidine-rich protein 2: HRP2; Lactate dehydrogenase: LDH; Polymerase chain reaction: PCR; Rapid diagnostic tests: RDT; World Health Organization: WHO.
